# Raspberry as a Source for the Development of *Drosophila suzukii* Attractants: Laboratory and Commercial Polytunnel Trials

**DOI:** 10.3390/insects10050137

**Published:** 2019-05-10

**Authors:** Rodrigo Lasa, Ricardo A. Toledo-Hernández, Douglas Rodríguez, Trevor Williams

**Affiliations:** 1 Instituto de Ecología AC, Xalapa, Veracruz 91070, Mexico; 2 Departamento de Investigación Aplicada, Driscoll’s Operaciones, Zapopan, Jalisco 45800, Mexico; ricardo.toledo@driscolls.com (R.A.T.-H.); douglas.rodriguez@driscolls.com (D.R.)

**Keywords:** fermented raspberry pulp, sucrose, apple cider vinegar, fly captures, SuzukiiTrap, Z-Kinol, spotted wing drosophila, SWD

## Abstract

Several commercial products and home-made attractants have been developed for monitoring and mass-trapping of the spotted wing drosophila, *Drosophila suzukii*. Growers in Mexico have adopted an attractant based on a fermenting mixture of raspberry pulp and sucrose, with anecdotally promising results. We compared the capture rates of traps baited with raspberry pulp + sucrose with captures from a range of alternative attractants. Raspberry pulp alone or with sucrose was more attractive than apple cider vinegar (ACV) or SuzukiiTrap and similar to baker’s yeast + sucrose in laboratory cage studies. Synthetic raspberry aroma (0.1–10% concentration), in water or mixed with ACV, did not improve capture rates in the laboratory. Traps baited with raspberry + sucrose or ACV had similar captures of *D. suzukii* in raspberry or blackberry polytunnels in Michoacán, Mexico. Raspberry + sucrose baited traps captured significantly higher numbers of other drosophilid species, leading to higher total numbers of captured flies (all species), which may explain why Mexican growers favor the raspberry-based attractant. The commercial products SuzukiiTrap and Z-Kinol had lower captures than ACV in polytunnels, although SuzukiiTrap had the highest selectivity in captures of *D. suzukii* (81% of flies captured). A two-component trap (2C trap) baited with ACV + ethanol as the drowning solution and raspberry pulp + sucrose or baker’s yeast + sucrose in a ventilated tube device was markedly more effective than the trap currently used by growers. We conclude that raspberry pulp + sucrose is as effective for the attraction of *D. suzukii* as ACV under commercial polytunnel conditions. The 2C trap performed better than the transparent cup trap currently used by berry producers in Mexico.

## 1. Introduction

The spotted wing drosophila, *Drosophila suzukii* (Matsumura) (Diptera: Drosophilidae), is an invasive insect that has spread around the world over the last decade [1]. This fly has become a major pest of wild and cultivated berry crops in Europe and the Americas [2,3], particularly in blackberry, raspberry, cherry, strawberry, and blueberry crops [4,5,6,7], although many other soft fruit species such as guava, grape, and cranberry may also be attacked [8,9,10]. Unlike most species of *Drosophila*, in which females oviposit on overripe and rotten fruit, the serrated ovipositor of *D. suzukii* enables females to oviposit within developing fruit [11]. Larvae then hatch and develop inside the fruit, rendering it unmarketable [5].

This pest arrived in Mexico in 2011 [12], and growers quickly established programs to detect and monitor the presence and abundance of fly infestation in greenhouses and polytunnels in the main berry production areas of the country. Traps are maintained following trapping protocols developed by Mexico’s Ministry of Agriculture, Rural Development, Fisheries and Food (SAGARPA). The SAGARPA-approved trap requires the use of a one-liter perforated transparent cup baited with apple cider vinegar (ACV) [13]. ACV has been widely used as an attractant for *D. suzukii* as it is cheap and readily available [14]. Mixtures of ACV with wine [15], or fermentation products, including yeasts, can significantly increase the response of *D. suzukii* to baited traps compared to traps baited with ACV alone [16,17,18]. Commercial attractants have also been developed for monitoring and mass-trapping of *D. suzukii* [18,19], including SuzukiiTrap (a mixture of organic acids and peptides) and Z-Kinol (a mixture of kairomones based on fruit volatiles). In addition, a two-component trap (named 2C trap) has been developed by us and consists of a perforated red and black plastic cup containing ACV + ethanol drowning solution and an additional ventilated plastic tube, suspended from the trap lid, containing a fermenting mixture of sucrose + baker’s yeast [20].

Tests on fly responses to host volatile compounds have indicated that raspberry fruit volatiles can be significantly more attractive to *D. suzukii* than strawberry, blueberry, or cherry volatiles [21]. At harvest, it is common that a fraction of the raspberry fruit crumbles when picked making them unmarketable [22]. Some growers in Mexico are currently using unmarketable raspberries, crushed and mixed with sucrose and water, to produce a fermenting lure that they report to be effective for trapping *D. suzukii* (R.A.T.H., pers. obs.).

To determine whether raspberry pulp could be used as a cheap and effective attractant, we compared attraction to raspberry pulp with that of a synthetic aroma, commercial lures, and ACV in laboratory cage experiments. Finally, the capture and selectivity of raspberry pulp + sucrose was compared with that of commercial lures using the SAGARPA and 2C trap designs under polytunnel conditions in the principal berry-producing region of Mexico.

## 2. Materials and Methods

### 2.1. Insect Colony and Fruits

A laboratory colony of *D. suzukii* was started in an insectary at the Instituto de Ecología AC, Xalapa, Veracruz, Mexico, using adults that emerged from naturally-infested wild blackberry, *Rubus fruticosus* L., collected at Xico, Veracruz, (19°25′59″ N; 97°1′59″ W, 1385 m altitude) in June 2015 and refreshed in November 2017 with adults from red guava, *Psidium cattleianum* Sabine, in Rancho Viejo, Veracruz (19°31′17″ N; 96°59′06″ W, 1434 m altitude). Adults were allowed to oviposit in a cornmeal-based artificial diet [23], dispensed into 300 mL plastic cups and covered with fine nylon gauze. The colony was maintained at 24 ± 1 °C, 60 ± 10% relative humidity (RH) and 12:12 h (L:D) photoperiod with a light intensity of 3500–4500 lux. Females flies used in laboratory tests were 5–7 days old, had been kept together with males in cages since emergence and were assumed to be mated. For laboratory tests, all performed in 2016 and 2017, raspberries (Driscoll’s, Jalisco, Mexico) were bought from a local supermarket. Unmarketable crumbly raspberry fruit were obtained directly from Driscoll’s polytunnels (Jacona, Michoacán, Mexico).

### 2.2. Lures

Apple cider vinegar (ACV) (La Costeña, 5% acidity, Ecatepec, Mexico) was used for laboratory tests, whereas ACV (Clemente Jacques, 5% acidity, Sabormex de México, Puebla, Mexico) was used for polytunnel trials because it was more readily available in the berry-growing areas of Michoacán state, Mexico. Traps baited with these vinegars captured nearly identical numbers of flies in laboratory tests (data not shown). Synthetic raspberry aroma (Saborex de México, Querétaro, Mexico) was used for laboratory experiments. Two commercial lures for *D. suzukii* were: (i) SuzukiiTrap (Bioibérica, Barcelona, Spain), a mixture of organic acids and peptides, and (ii) Z-Kinol (Squid Biological and Pheromones, Texcoco, Mexico) a kairomone attractant comprising a mixture of ethanoic acid, hydroxyl alcohols, thiol-alcohols, and ketones. Baker’s yeast (*Saccharomyces cerevisiae* Meyen ex E.C. Hansen) was obtained as a dry commercial product (Tredi-Pan, Safmex SA de CV, Toluca, Mexico).

### 2.3. Traps

**Figure 1:**
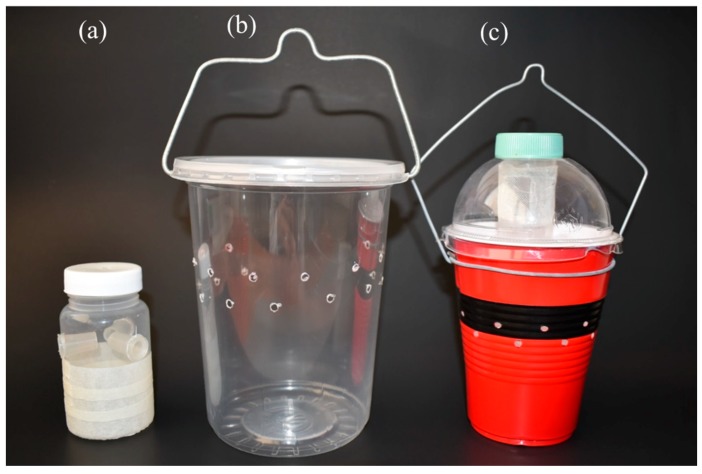
Trap models used in experiments: (**a**) handmade trap (120 mL capacity) used in laboratory cage experiments, (**b**) SAGARPA trap (one-liter capacity), (**c**) 2C trap comprising a cup with drowning solution and a ventilated tube device in the lid.

For laboratory cage tests, small traps were constructed from 120 mL plastic cups (35 mm diameter, 87 mm height) with three equidistant lateral holes through which translucent conical tubes (9 mm external diameter, 6 mm internal diameter, 20 mm deep) were inserted to reduce fly escape once inside the trap. Holes were placed at 45 mm from the base. The lower half of the cup was covered with cream-colored paper tape to facilitate fly landing on the surface of the trap (Figure 1a). For polytunnel tests, two different trap models were used: (i) a clear 1 l capacity transparent plastic cup with a flat lid (Plásticos Adheribles del Bajio, León, Mexico), with 10 lateral holes of 3.2 mm diameter (Figure 1b), recommended for monitoring *D. suzukii* in Mexico by SAGARPA, hereafter called SAGARPA trap and (ii) an opaque red polyethylene cup of 473 mL capacity (Walmart de México, Santa Cruz Acayucan, Mexico) with 20 holes of 3.2 mm diameter in two rows (10 equidistant holes per row) two-thirds of the way up the sides of the cup (Figure 1c). The cup is sealed with a dome-shaped lid in which a 50 mL capacity ventilated plastic centrifuge tube was inserted, hereafter called 2C trap, described in detail elsewhere [20]. A fermentation mixture placed in the ventilated tube device produces volatiles that are released into the headspace in the 2C trap and mix with volatiles from the drowning solution. This mixture of volatiles is then released through the lateral holes in the trap (Figure 1c).

### 2.4. Laboratory Cage Trails

#### 2.4.1. Attraction to Raspberry Pulp

Two independent multiple-choice tests were performed to evaluate the attraction of *D. suzukii* to raspberry pulp. In a first experiment, 140 g of raspberry fruits were crushed to a pulp in a ceramic mortar and 3 g samples of pulp were placed in 30 mL plastic cups and frozen until use. Small cup traps of 120 mL (described in the previous section) were baited with one of four different treatments: i) 3 g of defrosted raspberry pulp placed on a piece of cotton, ii) 3 mL ACV on a piece of cotton, iii) 3 g of defrosted raspberry pulp on a piece of cotton + 3 mL ACV dispensed on an additional piece of cotton inside the same trap, and iv) 3 mL water on a piece of cotton. Fruit pulp was defrosted 1 h prior to use.

One trap from each treatment was placed simultaneously at the corners of four acrylic cages (30 cm × 30 cm × 30 cm) with 0.2 mm nylon mesh walls. Traps were initially positioned at random in the first cage and were then placed at all the remaining positions in the other three cages. Forty non-starved flies (20 females and 20 males), between 5 and 7 days old, were released inside the cage at 10:00 am. The following day, 23 h after the flies had been released, traps were removed from cages and flies were killed by freezing. The flies captured in each trap were counted and sorted by sex. The remaining flies inside the cage were discarded. All traps were subsequently rotated clockwise by one position (one corner of the cage) for each new replicate. Treatments were evaluated simultaneously in four different cages, on four occasions, giving a total of 16 replicates.

A second experiment was performed to evaluate attraction to a raspberry pulp + sucrose solution mixture, used by berry producers in Mexico. This mixture was compared with other attractants previously reported to be attractive to *D. suzukii*. Treatments were: i) 20 mL raspberry pulp + sucrose solution (a mixture of 100 g of raspberry pulp + 5 g sucrose + 50 mL water that had been filtered through a 0.2 mm nylon mesh), ii) 20 mL *S. cerevisiae* + sucrose (0.4 g dry baker’s yeast + 1.1 g sucrose + 20 mL water), iii) 20 mL ACV and, iv) 20 mL SuzukiiTrap. Lures were placed in 120 mL cup traps and were maintained under laboratory conditions (24 °C) for 18–20 h prior to use. All treatments included 10 µL Tween 80 to reduce the surface tension of the liquids and improve the likelihood of fly drowning. Traps were initially placed at random in the corners of acrylic cages and the experiment was conducted following the methodology described above (total 16 replicates). After each 23 h period, the flies captured in each trap were killed by freezing, counted, and sorted by sex and the remaining flies inside the cage were discarded.

#### 2.4.2. Attraction to Synthetic Raspberry Aroma

Three independent tests were performed to evaluate the attraction of *D. suzukii* to diluted food quality raspberry aroma. In the first experiment, four treatments were compared: i) ACV as a reference treatment, ii) 1% raspberry aroma in water, iii) 5% raspberry aroma in water, and iv) 10% raspberry aroma in water. All treatments were prepared in a volume of 20 mL per trap and included 10 µL of Tween 80. The same 120 mL trap and experimental acrylic cages were used, and the experiments were performed as described above, except that the experiment was performed twice using different batches of flies, resulting in 8 replicates in total.

Due to the low attraction to synthetic raspberry aroma in water, a second experiment was performed in which raspberry aroma was diluted in ACV. Four treatments were compared: i) ACV as a reference treatment, ii) 1% raspberry aroma diluted in ACV, iii) 5% raspberry aroma in ACV, and iv) 10% raspberry aroma in ACV. All treatments were prepared in a volume of 20 mL per trap and included 10 µL of Tween 80. The methodology of the experiment was the same as described above with a total of 8 replicates.

The third experiment was performed to evaluate a lower concentration of raspberry aroma in ACV. Two treatments were compared: i) ACV, and ii) ACV + 0.1% raspberry aroma. As before, both treatments were prepared in a volume of 20 mL per trap and included 10 µL of Tween 80. The methodology of the experiment was the same as described above with a total of 8 replicates.

### 2.5. Commercial Polytunnel Trials

#### 2.5.1. Capture and Selectivity of Raspberry Pulp and Other Products

In a first trial, raspberry pulp + sucrose attractant was compared with ACV and two commercial lures in three polytunnels growing raspberries and two polytunnels growing blackberries. SAGARPA traps contained one of the following four attractants: (i) 150 mL of raspberry pulp + sucrose solution described in the laboratory cage studies, (ii) 150 mL of ACV as a reference treatment, (iii) 150 mL of SuzukiiTrap, and (iv) a polyethylene sachet of Z-Kinol attached under the lid of the trap with 250 mL of water as the drowning solution. All lures, except SuzukiiTrap, contain a drop of odorless detergent (ML-100, Beta Procesos, Celaya, Guanajuato, Mexico) to improve the likelihood of fly drowning. Raspberry pulp + sucrose solution was prepared every week, filtered through a 0.2 mm nylon mesh and kept at room temperature for 24 h before use in the polytunnel trial. All attractants were evaluated in a similar colorless plastic SAGARPA trap.

**Figure 2:**
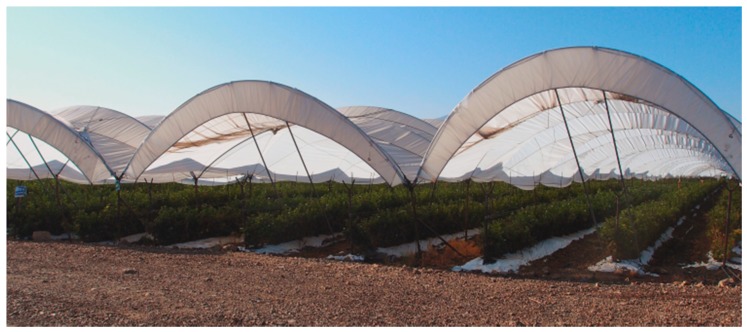
Open-sided commercial polytunnels used for experiments on trap-lure combinations for capture of *D. suzukii* in berry crops in Michoacán, Mexico.

Trials were performed during the fruiting season of April–May 2016 in three independent commercial raspberry polytunnels, named A–C, in the berry-producing region of Michoacán, Mexico, located at A (19°40′5″ N, 102°25′42″ W), B (19°53′15″ N, 102°04′56″ W), and C (19°32′13″ N, 102°27′51″ W), and two blackberry polytunnels, named D and E, located at D (19°53′8″ N, 102°09′24″ W) and E (19°22′8″ N, 102°21′48″ W). Polytunnels were open-sided, 6.6 m in height, varied from 60 to 140 m in length and were covered with white polythene (Figure 2). Each set of polytunnels was divided into three blocks of 150 m × 20 m (~0.3 ha/block). All attractants were placed at random within a block, on metal stakes at a height of 1.5 m above the ground at a distance of at least 10 m from the edge of the polytunnel and 30 m distance between traps. The traps were inspected at 7-day intervals. Every week, at the inspection, ACV and raspberry pulp were replaced with freshly-prepared lures, whereas Z-Kinol and SuzukiiTrap were replaced only after 4 consecutive weeks in the field, following manufacturer’s recommendations. At each inspection, captured flies were placed in 70% ethanol. All attractants were subsequently rotated clockwise by one position for each new replicate during 8 consecutive weeks of the study (twice per position). Fly samples in ethanol were taken to the laboratory where they were counted, identified, and classified into one of three categories: *D. suzukii*, *Zaprionus indianus* Gupta (another exotic pestiferous drosophilid) [24], or other species of drosophilids.

#### 2.5.2. Evaluation of SAGARPA and 2C Trap-Lure Combinations

As previous studies in the berry-growing region of Mexico revealed a high attraction to the 2C trap [18], an additional test was performed in a blackberry polytunnel (~4 ha) in fruit production during October 2017 to determine whether the performance of the 2C trap could be improved by incorporating raspberry pulp + sucrose. The SAGARPA trap baited with ACV or raspberry pulp + sucrose was included as a reference. For this, a trial was performed in Tangancícuaro, Michoacán (19°54′25″ N, 102°12′15″ W; altitude 1730 m) to evaluate four trap-lure combinations: i) 2C trap baited with 150 mL mixture of ACV + 10% ethanol + 10 µL Tween 80 as the drowning solution, and the additional tube device containing a freshly-prepared mixture of 0.47 g baker’s yeast and 1.1 g sucrose and 20 mL of water, as tested previously [18], ii) an identical 2C trap containing 150 mL mixture of ACV + 10% ethanol + 10 µL Tween 80 as the drowning solution but with the tube device containing 20 mL of raspberry pulp + sucrose solution, as described in laboratory cage tests, iii) SAGARPA trap containing 150 mL ACV, and iv) SAGARPA trap containing 150 mL raspberry pulp + sucrose solution.

As in the previous trial, traps were randomly placed at least 10 m from the edge of the polytunnel, 30 m apart and at a height of 1.5–1.6 m in five different blocks. The traps were sampled and rotated at weekly intervals for 5 weeks. Each week, at inspection, all lures were renewed. Captured flies were placed in 70% ethanol and were taken to the laboratory where they were counted, identified and classified as *D. suzukii*, *Z. indianus*, or other species of drosophilids.

### 2.6. Statistical Analysis

Mean numbers of flies captured in laboratory tests were subjected to a two-way analysis of variance (ANOVA) with attractant and sex as factors. Means were compared by the Tukey test (*P* ≤ 0.05). If required, a √ (*x* + 1) transformation was applied to achieve homoscedasticity prior to analysis. Mean captures in traps baited with ACV with and without 0.1% raspberry aroma were compared by t-test. Numbers of trapped flies in polytunnel trials were averaged across traps for each day of the trial to generate flies/trap/day values prior to analysis. Flies/trap/day values were analyzed by fitting generalized linear models (GLM) with a negative binomial distribution, followed by Bonferroni mean separation. The significance of changes in GLM model deviance were determined with reference to *χ*^2^ statistics.

Trap selectivity, considered as the percentage of *D. suzukii* flies trapped among total drosophilids could not be normalized by transformation and was compared among treatments in both polytunnel experiments by Kruskal–Wallis test, followed by Dwass–Steel–Chritchlow–Fisher (DSCF) pairwise comparisons [25]. All analyses were performed using the R-based program Jamovi v.0.9.5.17 [26].

## 3. Results

### 3.1. Laboratory Cage Trails

#### 3.1.1. Attraction to Raspberry Pulp

**Figure 3:**
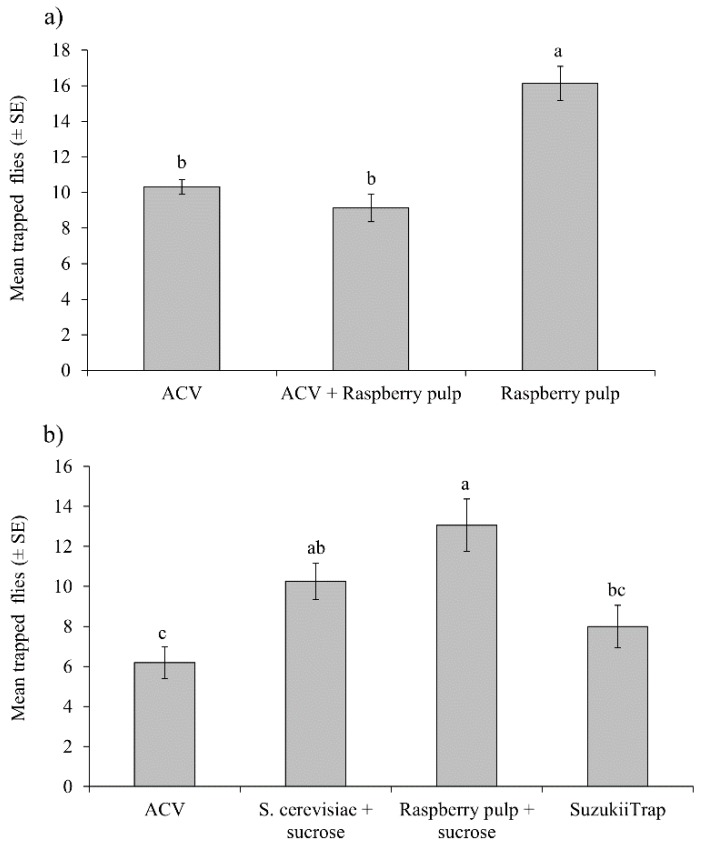
Mean numbers of *D. suzukii* (males and females) trapped in different treatments in two independent experiments: (**a**) comparison of raspberry pulp and ACV, and (**b**) comparison of ACV, baker’s yeast + sucrose, raspberry pulp + sucrose, or the commercial product SuzukiiTrap. Columns labelled with the same letter did not differ significantly (Tukey, *p* > 0.05).

The mean capture of flies was significantly higher for raspberry pulp than for ACV or a mixture of ACV + raspberry pulp (*F* = 13.02; df = 2,90; *p* < 0.001) (Figure 3a). No significant differences were observed in captures according to sex (*F* = 0.001; df = 1,90; *p* = 0.972), or the interaction of treatment*sex (*F* = 2.09; df = 2,90; *p* = 0.130). Captures of flies in control traps containing water were almost zero, so that this treatment was not included in this analysis, or in any of the subsequent laboratory or polytunnel trails.

In a separate experiment, traps baited with a mixture of raspberry pulp + sucrose or baker’s yeast + sucrose had the highest mean captures of flies (Figure 3b), whereas captures of flies were lowest in traps baited with ACV and were intermediate in SuzukiiTrap-baited traps (*F* = 11.42; df = 3,120; *p* < 0.001). No significant differences were observed for sex (*F* = 0.86; df = 1,120; p=0.356), or the interaction of treatment*sex (*F* = 1.15; df = 2,120; p = 0.332).

#### 3.1.2. Attraction to Synthetic Raspberry Aroma

**Figure 4:**
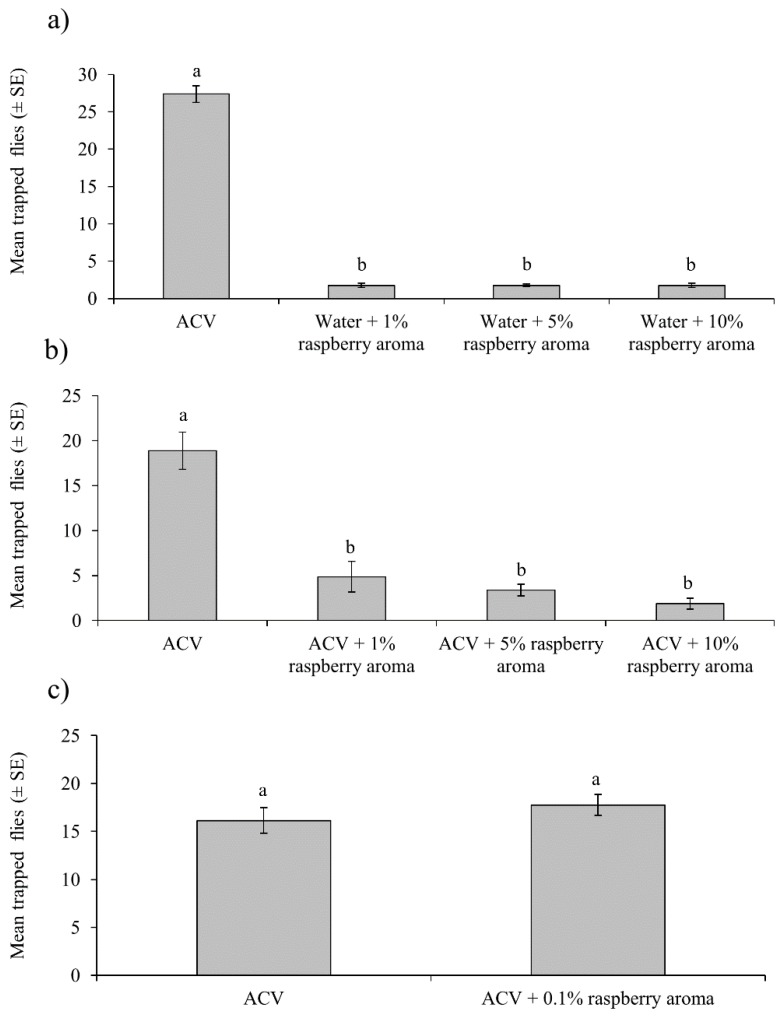
Mean numbers of *D. suzukii* (males and females) trapped in ACV or different treatments that contained synthetic raspberry aroma in water (**a**), in mixtures with ACV (**b**) or included at low concentration (0.1%) in ACV (**c**). Columns labeled with the same letter did not differ significantly (Tukey, *p* > 0.05).

The mean capture of flies in ACV-baited traps was significantly higher than any of the raspberry aroma treatments (*F* = 23.89; df = 3,56; *p* < 0.001) (Figure 4a). Captures did not differ significantly according to sex (*F* = 0.16; df = 1,56; *p* = 0.690), or the interaction of treatment*sex (*F* = 0.770; df = 3,56; *p* = 0.516).

In the following experiment, the capture of flies was significantly higher for ACV traps than for any of the mixtures of ACV + raspberry aroma (*F* = 10.86; df = 3,56; *p* < 0.001) (Figure 4b), irrespective of sex (*F* = 0.15; df = 1,56; *p* = 0.705), or the interaction of treatment*sex (*F* = 0.43; df = 3,56; *p* = 0.731).

In the final laboratory experiment, when the concentration of raspberry aroma was reduced to 0.1%, the mean capture of flies was similar for ACV alone and ACV + 0.1% raspberry aroma (*t* = 0.94; df = 14; *p* = 0.365) (Figure 4c).

### 3.2. Polytunnel Trials

#### 3.2.1. Capture and Selectivity of Raspberry Pulp and Other Products

A total of 15,740 drosophilid flies were trapped in the first trial, of which 8779 (56%) were *D. suzukii*, 2299 (15%) were *Z. indianus*, and 4662 (29%) were other drosophilid species.

**Figure 5:**
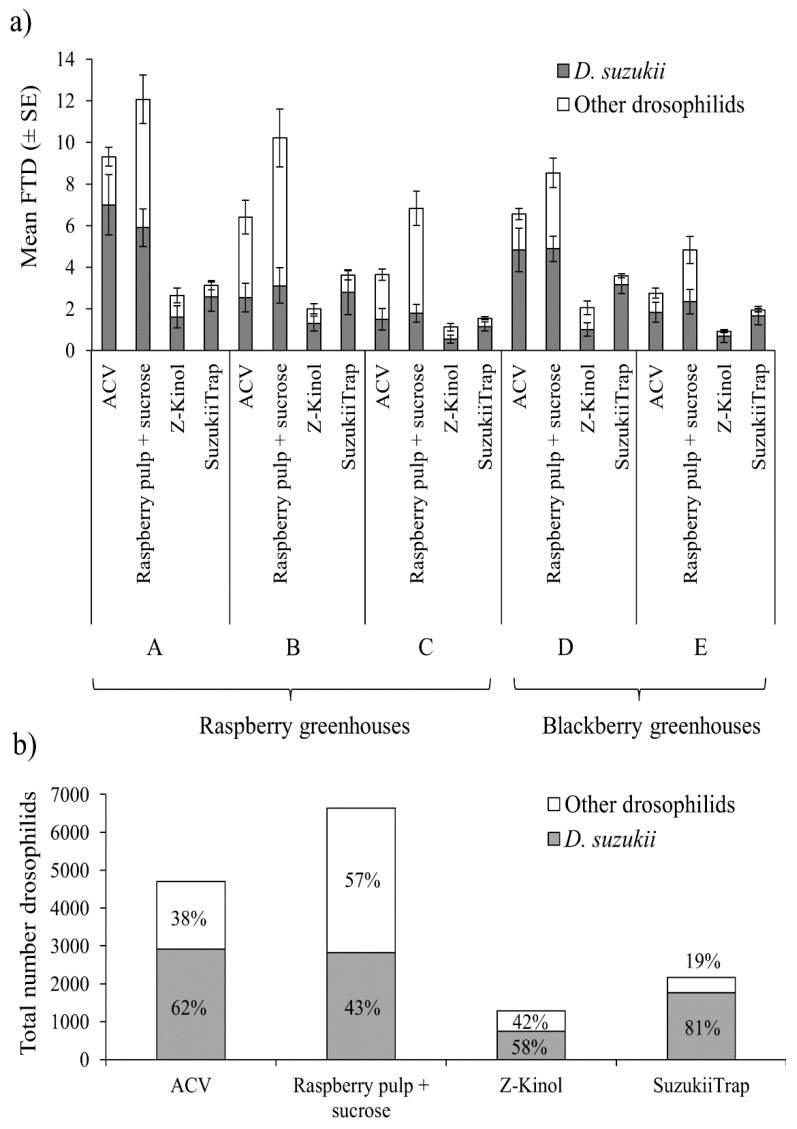
(**a**) Mean numbers of flies/trap/day (FTD ± SE) for *D. suzukii* (grey columns) and other drosophilid species (white columns) trapped using different attractants in three commercial polytunnels of raspberry (A–C) and two of blackberry (D,E). (**b**) Total numbers of *D. suzukii* flies (grey columns) and other drosophilid species (white columns), that were trapped for each attractant across all polytunnel trials shown in (**a**). Values within columns indicate percentage species composition.

Considering *D. suzukii* alone, the mean flies/trap/day values differed significantly among attractants (*χ*^2^ = 71.97; df = 3; *p* < 0.001), but did not differ significantly between raspberry and blackberry polytunnels (*χ*^2^ = 0.40; df = 1; *p* = 0.526), or the interaction of attractant*crop (*χ*^2^ = 1.50; df = 3; *p* = 0.526) (Figure 5a). However, for *D. suzukii*, no significant differences were observed between ACV and raspberry pulp + sucrose in the mean flies/trap/day value, although both attractants captured significantly more *D. suzukii* flies than SuzukiiTrap, whereas the least effective attractant was Z-Kinol.

The mean total number of drosophilid flies (all species including *D. suzukii*) trapped per trap per day also differed significantly among attractants (*χ*^2^ = 165.30; df = 3; *p* < 0.001) and crop (raspberry vs. blackberry) (*χ*^2^ = 6.49; df = 1; *p* = 0.011), but the interaction of attractant*crop was not significant (*χ*^2^ = 2.45; df = 3; *p* = 0.484) (Figure 5a). The highest number of total drosophilids was captured in traps baited with raspberry pulp + sucrose, followed by ACV, SuzukiiTrap and Z-Kinol (Figure 5b).

The selectivity of attractants in terms of the species composition of captured flies also differed significantly (*χ*^2^ = 81.10; df = 3; *p* < 0.001). Overall, SuzukiiTrap was the most selective lure with 81% of captured flies being *D. suzukii* and raspberry pulp + sucrose was the least selective lure with 43% of captures being *D. suzukii* (Figure 5b). ACV and Z-Kinol had intermediate levels of selectivity, with 62 and 58% of *D. suzukii* among the captured flies, respectively. However, as is clear in Figure 5b, the higher selectivity of SuzukiiTrap was not associated with the highest numbers of *D. suzukii* flies captured, which occurred in ACV and raspberry pulp + sucrose baited traps.

#### 3.2.2. Evaluation of SAGARPA and 2C Trap-Lure Combinations

A total of 11,753 drosophilid flies were trapped in the blackberry polytunnel of which 8367 (71%) were *D. suzukii* and 3386 (29%) were other species of drosophilids, with just 93 individuals of *Z. indianus*.

**Figure 6:**
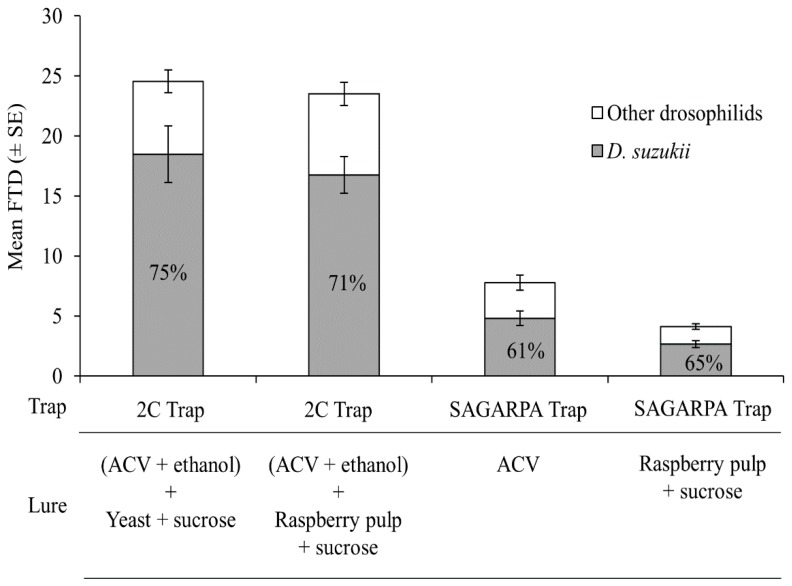
Mean numbers of flies/trap/day (FTD) for *D. suzukii* (grey columns) and other drosophilid species (white columns) trapped in 2C traps and SAGARPA traps baited with different lure combinations that were evaluated in a commercial polytunnel blackberry production system.

The total number of drosophilid flies/trap/day differed significantly among trap-lure combinations (*χ*^2^ = 181.0; df = 3; *p* < 0.001), as did the mean flies/trap/day values for *D. suzukii* (*χ*^2^ = 180.0; df = 3; *p* < 0.001) (Figure 6). Mean flies/trap/day values for total drosophilids and for *D. suzukii* flies alone in the 2C trap that had ACV + ethanol as the drowning solution, were similar when baited with baker’s yeast + sucrose or with raspberry pulp + sucrose in the upper tube device. The 2C trap also had markedly higher numbers of captures than the SAGARPA trap baited with ACV or raspberry pulp + sucrose (Figure 6). Mean flies/trap/day values for total drosophilids or D. suzukii alone were both significantly higher in ACV-baited SAGARPA traps than for the same traps baited with raspberry pulp + sucrose.

The selectivity of all trap-lure combinations in the capture of *D. suzukii* and other drosophilids in this experiment was similar, with 61–75% of *D. suzukii* flies and 25–39% of other drosophilid species across all trap-lure combinations (*χ*^2^ = 3.13; df = 4; *p* < 0.530).

## 4. Discussion

Laboratory cage experiments showed a significantly higher attraction of both sexes of *D. suzukii* to raspberry pulp over ACV. Placing raspberry pulp and ACV on separate cotton pads in the same trap did not improve attraction compared to ACV alone. Raspberry pulp + sucrose was as effective as an attractant as a fermenting mixture of *S. cerevisiae* + sucrose under laboratory conditions, and more effective than ACV alone or the commercial lure SuzukiiTrap. Mixtures of synthetic raspberry aroma and water, or aroma and ACV, markedly reduced attraction when compared with ACV alone (Figure 4a–c). Despite the clear attraction of *D. suzukii* to raspberry pulp over ACV under laboratory conditions, no significant differences were observed in capture rates (flies/trap/day) of *D. suzukii* when both attractants were compared in commercial polytunnels.

In polytunnel trials, raspberry pulp + sucrose and ACV attracted similar numbers of *D. suzukii*, whereas SuzukiiTrap captured significantly fewer flies followed by Z-Kinol. Differences in attraction in laboratory and polytunnel trials could be due to the fermentation conditions of the raspberry + sucrose mixture over the course of each week, compared to the 24 h period of laboratory cage observations. For example, flies or other insects trapped during each week of the trial were likely to have carried different yeast or bacterial species on their bodies [27,28,29,30,31], that could have contaminated the sucrose-enriched raspberry pulp, changing the mixture of volatiles released and influencing its attractiveness to *D. suzukii*.

Captures of *D. suzukii* were similar in raspberry and blackberry crops, likely reflecting the preferred host status of these species for this pest. However, when the total captures of drosophilids were analyzed (Figure 5b), two findings became clear. First, that raspberry pulp + sucrose consistently captured a greater number of all drosophilid species than any of the other attractants and second, that for capturing *D. suzukii* raspberry pulp + sucrose and ACV were almost identical in their effectiveness. The high rate of captures in raspberry + sucrose baited traps is likely to be responsible for the belief of Mexican berry growers that raspberry + sucrose in a better attractant for *D. suzukii* than ACV. However, the lower selectivity of raspberry + sucrose, in which 43% of captures were *D. suzukii*, meant that this attractant and ACV were almost identical in their ability to capture *D. suzukii*.

In the second polytunnel experiment, in a blackberry crop, the raspberry pulp + sucrose mixture was less attractive to *D. suzukii* than ACV when used to bait the SAGARPA trap. The second polytunnel trial also highlighted the efficiency of the 2C trap, baited with a combination of ACV + 10% ethanol as the drowning solution, and fermenting mixtures of sugar + yeast or raspberry pulp + sucrose placed in a ventilated tube device placed through the dome-shaped lid (Figure 1c). In this case, as the fermenting mixtures were placed in the ventilated tube, trapped flies or other small insects could not contaminate the mixtures with insect-associated microorganisms, so that the release of volatiles during fermentation was probably due to the microbiota present on the raspberry fruit, or the fermentation of *S. cerevisiae* alone. Previous studies have also highlighted the performance of the 2C trap with respect to other commercial trap-attractant combinations [20].

Adult females of *D. suzukii* use volatiles released from berry fruits or from fermented baits to select oviposition and feeding sites [21,32,33,34]. Raspberry fruits have been reported to be highly attractive to adult female and male *D. suzukii* [21] and were preferred for oviposition in choice tests [35]. Indeed, when developing a preference ranking index, Bellamy et al. [36] ranked raspberry as the most preferred host followed by blackberries and strawberries. That said, the synthetic raspberry aroma in water or mixed with ACV failed to increase or even reduced captures in laboratory cage trials. It is quite possible that the olfactory cues of synthetic raspberry aroma are perceived differently by flies and humans. However, synthetic strawberry aroma mixed with fermenting yeast + sucrose solution improved attraction of *D. suzukii* females to traps placed in Asian fruit markets [37].

SuzukiiTrap and Z-Kinol were used as reference commercial attractants because they are currently available and used in berry production polytunnels in Mexico. SuzukiiTrap was the most selective attractant for *D. suzukii*, as has been reported previously [18,19], but this product captured lower overall numbers of *D. suzukii* than ACV or raspberry pulp + sucrose. This finding is at variance with studies by others who reported SuzukiiTrap to be more effective than ACV in different settings in the United States and Italy [18,19]. In contrast, to date, there are no published comparisons of the performance of Z-Kinol in Mexico or elsewhere. This product had low capture rates in both raspberry and blackberry polytunnels in our study. In the absence of any published comparative studies, it is difficult to suggest the reasons behind the low capture rates that we observed with this product.

Highly attractive and selective attractants are desirable for both pest monitoring and mass-trapping programs, although lures that combine these characteristics have not been developed yet. In general, most attractants that captured high numbers of *D. suzukii*, also trapped high numbers of other drosophilid species [17,18,19,35]. The efficacy of trap-attractant combinations can also vary across crops and locations [14,35,38]. Consequently, the decision to use a particular trap-attractant combination often depends on cost, the crop, the weather conditions, and the desire to meet specific needs, such as selectivity or early detection, involved in pest monitoring or mass-trapping based pest management strategies.

## 5. Conclusions

We conclude that raspberry pulp + sucrose was an effective attractant for *D. suzukii* flies under laboratory and commercial polytunnel conditions. Although raspberry pulp + sucrose captured the highest number of drosophilids (all species), the lower selectivity of raspberry pulp + sucrose meant that capture rates of *D. suzukii* were similar to the rates observed in ACV-baited traps under polytunnel conditions. Traps baited with raspberry pulp + sucrose or ACV captured higher numbers of *D. suzukii* than the commercial products SuzukiiTrap or Z-Kinol.
